# Synthesis and Biological Evaluation of Imadazo[1,2-*a*]pyrazines as Anticancer and Antiviral Agents through Inhibition of CDK9 and Human Coronavirus

**DOI:** 10.3390/ph15070859

**Published:** 2022-07-13

**Authors:** Aisha A. Alsfouk, Hanan M. Alshibl, Bshra A. Alsfouk, Najla A. Altwaijry, Ebtehal S. Al-Abdullah

**Affiliations:** 1 Department of Pharmaceutical Sciences, College of Pharmacy, Princess Nourah Bint Abdulrahman University, Riyadh 11671, Saudi Arabia; baalsfouk@pnu.edu.sa (B.A.A.); naaltwaijry@pnu.edu.sa (N.A.A.); 2 Department of Pharmaceutical Chemistry, College of Pharmacy, King Saud University, Riyadh 11451, Saudi Arabia; halshibl@ksu.edu.sa (H.M.A.); ealabdullah@ksu.edu.sa (E.S.A.-A.)

**Keywords:** kinase inhibitor, cyclin-dependent kinase, anti-proliferative, COVID-19, SARS-CoV-2, HCoV-229E

## Abstract

In this work, novel imadazo[1,2-*a*]pyrazine derivatives were synthesized and evaluated as CDK9 inhibitors. The results of CDK9 assay showed that the derivatives with pyridin-4-yl in position 2 and benzyl in position 3 of imadazo[1,2-*a*]pyrazine **3c** displayed the most potent CDK9 inhibitory activity with IC_50_ of 0.16 µM. The anti-proliferative effect of the new compounds was examined against breast cancer (MCF7), colorectal cancer (HCT116), and chronic myelogenous leukaemia (K652) cell lines. The data of MTT assay showed that the cytotoxic effect of the inhibitors is correlated to their inhibitory activity against CDK9. Compound **3c** exhibited the most potent cytotoxicity effect with average IC_50_s of three cell lines of 6.66 µM. The drug likeness properties of **3c** were predicated in silico and demonstrated that **3c** have reasonable physiochemical and pharmacokinetic properties. Selected derivatives were assessed in antiviral assay against human coronavirus 229E. The results of this assay showed that the derivative with pyridin-4-yl in position 2 and cyclohexyl in position 3 of imadazo[1,2-*a*]pyrazine **3b** exhibited the most potent anti-coronaviral activity with IC_50_ of 56.96 µM and selectivity index of 7.14. The target predication result revealed that **3b** showed high affinity to protease enzyme. Docking studies of **3b** with COVID-19 main protease was conducted and showed good binding affinity, which confirmed the in vitro assay data.

## 1. Introduction

Cyclin-dependent kinase (CDK) is a family of enzymes that dimerize with specific proteins called cyclins. These CDK-cyclin heterodimers have crucial roles in gene transcription and cell cycle progression [1]. CDK9 is a member of the CDK family, which forms a heterodimer complex with cyclin T. This dimer is known as Positive Transaction Elongation Factor b (p-TEFB), which stimulates the transcriptional elongation by phosphorylation of RNA polymerase II at Ser2 and Ser5 [2,3].

There is sufficient evidence that supports the role of CDK9 as a molecular target for treatment of a number of malignancies including breast cancer, colorectal cancer, and acute myeloid leukaemia (AML) [4,5,6,7,8,9]. CDK9 conducts its oncogenic function through the regulation of anti-apoptotic agents such as Mc-1 and Myc and the promotion of cell proliferation [10,11].

**Figure 1:**
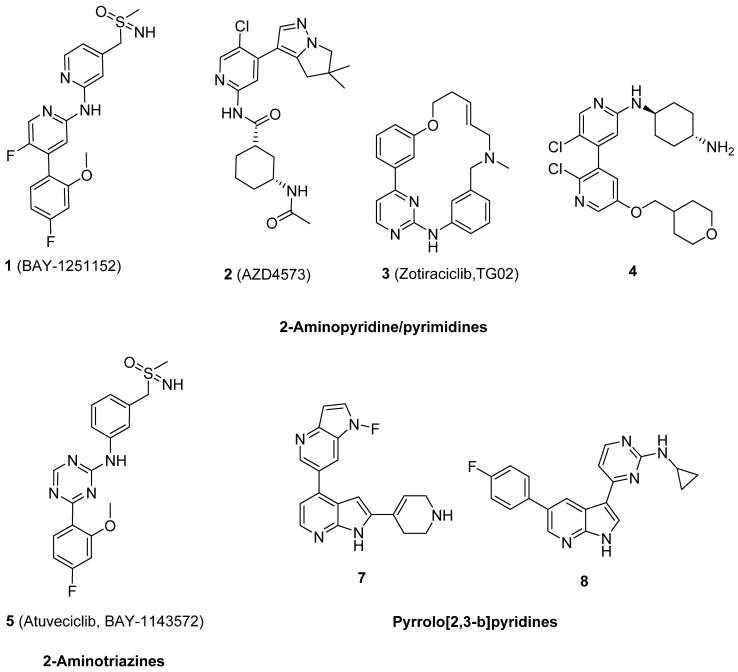
The main scaffolds of currently available clinical and patent CDK9 inhibitors.

Large pharmaceutical companies have developed a huge number of molecules as CDK9 inhibitors, and most of them are small molecules with good drug likeness properties and potent antitumor activity. Several of these were proceeded to clinical trials as anticancer drugs to treat various types of cancer [12,13,14,15,16,17,18,19,20]. The main chemical scaffolds of clinical and patent CDK9 inhibitors were focused on 2-aminopyridine/pyrimidines, such as BAY-1251152 **1**, AZD4573 **2**, Zotiraciclib (TG02 **3**), and **4**; 2-aminotriazines, such as **5** (Atuveciclib, BAY-1143572); and pyrrolo[2,3-b]pyridine, such as **6** and **7**, as shown in Figure 1 [21,22,23,24,25,26].

**Figure 2:**
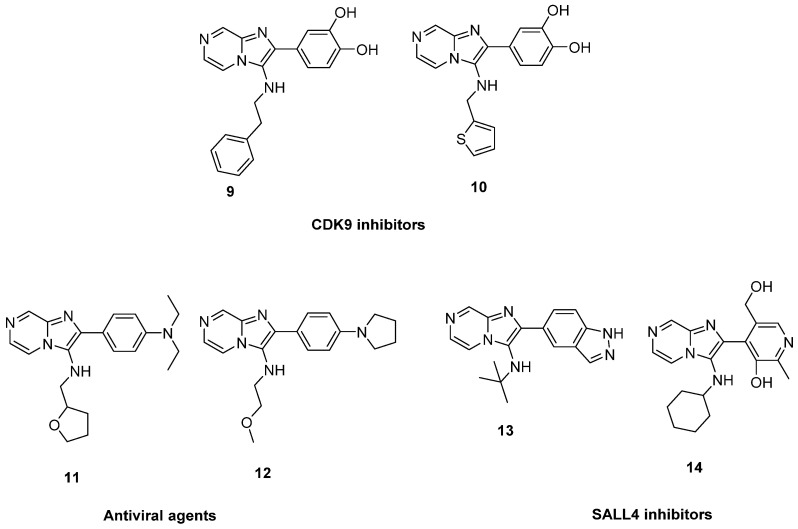
2-Phenylimidazo[1,2-*a*]pyrazin-3-amines reported in the literature.

In 2019, the imidazo[1,2-*a*]pyrazines **9** and **10** were identified by virtual screening from natural resources as CDK9 inhibitors with IC_50_ values of 7.88 and 5.12 µM, respectively, and evaluated as anticancer agents against breast cancer [27]. In a separate patent, compounds with 2-phenylimidazo[1,2-*a*]pyrazin-3-amine scaffold were reported as antiviral agent against influenza A virus such as **11** and **12** [28]. Compounds with similar structures such as **13** and **14** were reported in another patent as inhibitors of the transcriptional factor SALL4 and evaluated as anticancer agents against AML cell lines (Figure 2) [29].

In this study, imidazo[1,2-*a*]pyrazine was used as a scaffold for the preparation of CDK9 inhibitors and antiviral agents. Apart from the patent that reported the identification of two compounds having this scaffold by virtual screening from natural resources as inhibitors of CDK9 [27], no other published data, to the best of our knowledge, reports the development of imidazo[1,2-*a*]pyrazine as CDK9 inhibitors. Therefore, the rationale of choosing this scaffold is to develop a new class of CDK9 inhibitors bearing a different scaffold from those already extensively studied and currently available in the literature.

The aim of this study is to develop novel 2-phenylimidazo[1,2-*a*]pyrazin-3-amine as CDK9 inhibitors and examine their cytotoxicity as anticancer agents against a panel of cancer cell lines, study their binding mode, and evaluate of their drug-likeness properties in silico. Pathogenic human coronaviruses are species that cause respiratory diseases, appearing early in 2002 by the identification of SARS-CoV and recently by SARS-CoV-2, which caused the COVID-19 pandemic in 2019 [30]. There are a few pathogenic species of human coronaviruses such as 229E, OC43, and NL63 that cause mild diseases but share a similar homology sequence [31]. In this study, the antiviral activity of the new compounds was also evaluated against human coronavirus.

## 2. Results and Discussion

### 2.1. Chemistry

**Scheme 1:**
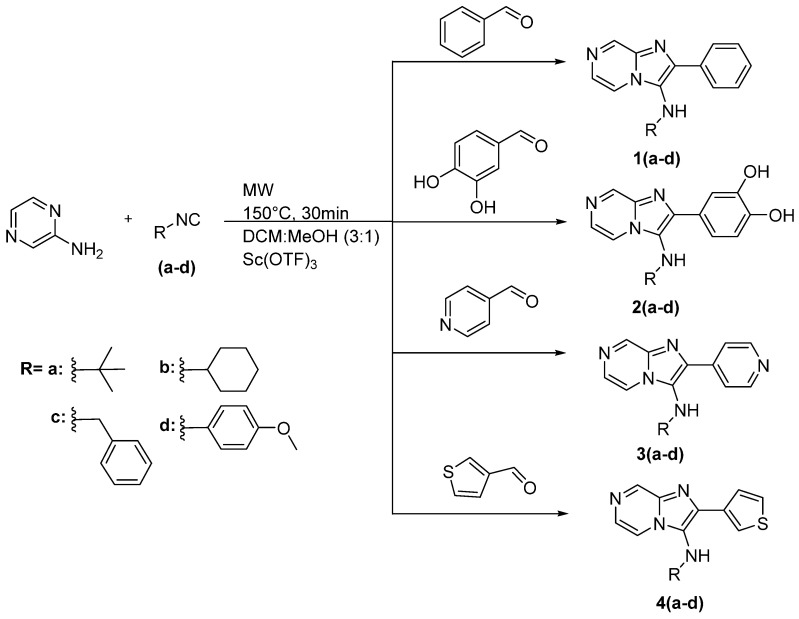
The synthetic approach used to synthesize the new compounds **1a**–**4d**.

The synthetic approach that was used to synthesize the novel compounds is described in Scheme 1. In this work, the new compounds were synthesized via one-pot multicomponent Groebke-Blackburn-Bienaymé reaction by reacting 2-aminopyrazine with various isocyanides and various aldehydes in the presence of scandium(III)-trifluoromethanesulfonate as catalyst and DCM:MeOH (3:1) as a solvent system. The reaction was carried out in a microwave under 150 °C temperature for 30 min. The products were purified by column chromatography using ethyl acetate: hexane.

The first serial of compounds **1(a**–**d)** have phenyl in position 2 of imadazo[1,2-*a*]pyrazine and various amines in position 3 including aliphatic, aromatic, cyclic, and acyclic amines. The yield of this series ranges from 84 to 96%. The second series **2(a**–**d)** with phenyl-3,4 diol in position 2 of imadazo[1,2-*a*]pyrazine and the yield ranged from 75 to 91%. In the third series **3(a**–**d)**, pyridin-4-yl is placed on position 2 of imadazo[1,2-*a*]pyrazine, and this series obtained an excellent yield 88–97%. The fourth series **4(a**–**d)** had thiophen-3-yl in position 2 of imadazo[1,2-*a*]pyrazine. These compounds were achieved in yield from 85% to 90%.

**Figure 3:**
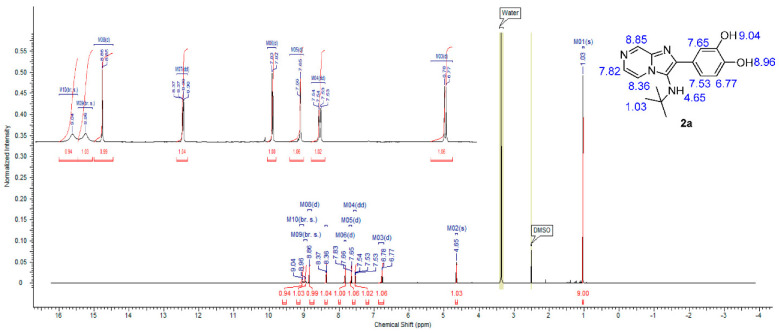
^1^H NMR spectrum of compound **2a**.

**Figure 4:**
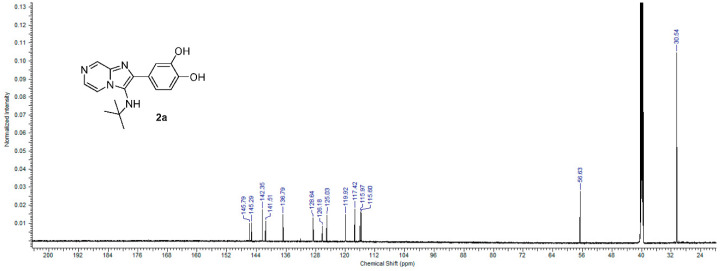
^13^C NMR spectrum of compound **2a**.

The formation of imadazo[1,2-*a*]pyrazine core was confirmed by ^1^H and ^13^C NMR spectra by appearances of three signals in aromatic region corresponding to pyrazine ring methylene protons in ^1^H NMR spectrum and six signals in the aromatic region, corresponding to imadazopyrazine carbons in ^13^C NMR spectrum in all compounds. ^1^H NMR spectrum for compound **2a**, for example, showed the appearance of two pairs of doublet at 7.82 ppm (d, *J* = 4.52 Hz, 1H) and 8.86 ppm (d, *J* = 1.29 Hz, 1H) in addition to the appearance of a doublet signal at 8.37 ppm (dd, *J* = 4.63, 1.40 Hz, 1H), representing the three methylene protons of imadazopyrazine core (Figure 3). A ^13^C NMR spectrum for compound **2a** showed the appearance of six aromatic signals at the region from 145 to 116 ppm, representing imadazopyrazine carbons, in addition to another six aromatic signals at the same region, representing the phenyl-3,4 diol ring at position 2 of imadazo[1,2-*a*]pyrazine. Two aliphatic carbon signals appeared at 56.63 and 30.54 ppm, representing the *tert*-butyl group attached to the amino group at position 3 of imadazo[1,2-*a*]pyrazine confirm the structure of **2a** (Figure 4).

### 2.2. Anti-Cancer Activity

#### 2.2.1. CDK9 Inhibitory Activity

Cyclin-dependent kinase-9 (CDK9) enzyme forms a heterodimer with cyclin T. This complex is known as Positive Transaction Elongation Factor b (p-TEFB), which has a crucial role as elongation factor during RNA transcription [2,3]. CDK9 phosphorylates C-terminus of RNA polymerase II [9,26].

There is compelling evidence to support the role of CDK9 as a molecular target for treatment of a number of malignancies via the regulation of anti-apoptotic proteins and the promotion of cancer initiation and progression [4,5,6,32,33,34].

In this study, all the newly synthesized compounds were assessed against CDK9 in a biochemical assay, and dinaciclib was used as a standard compound. Dinaciclib is a well-established Pan-CDKs inhibitor with low nM potency against CDK9 (literature IC_50_ of 4 nM [35,36]). The results are presented in Table 1 as IC_50_ (µM).

The data indicate that compounds with pyridin-4-yl at position 2 of imadazo[1,2-*a*]pyrazine **3(a**–**d)** displayed the most potent CDK9 inhibitory activity with sub µM IC_50_. Among them, **3c**, a compound with benzyl amine in position 3 of imadazo[1,2-*a*]pyrazine, was found as the most active derivative, with IC_50_ of 0.16 µM.

Furthermore, the other derivatives with benzyl amine in position 3 of imadazo[1,2-*a*]pyrazine, **2c** and **4c** were also exhibited good CDK9 inhibitory activity with IC_50_ of 0.31 and 0.71 µM, respectively.

On the other hand, compounds with 4-methoxyphenyl attached to the terminal amine at position 3 of imadazo[1,2-*a*]pyrazine such as **1d**, **2d**, and **4d** displayed weak inhibitory activity CDK9 (IC_50_ of 1.04, 2.03, and 3.16 µM, respectively).

#### 2.2.2. Cytotoxicity Activity

All synthesized compounds were evaluated for their cytotoxicity against breast cancer (MCF7), colorectal cancer (HCT116), and chronic myelogenous leukaemia (K652) cell lines using staurosporine as positive control in MTT assay. The results are shown in Table 2 as IC_50_ (µM).

The data of the MTT assay showed that the cytotoxic effect of the inhibitors is correlated to their inhibitory activity against CDK9. The most potent compounds in the biochemical assay against CDK9, for example, **3c**, **3a**, and **1b** (IC_50_ of 0.16, 0.26, and 0.25 µM, respectively), also exhibited the greatest cytotoxicity effect in the anti-proliferation assay (average IC_50_ of 6.66, 17.66, and 18.24 µM, respectively). Moreover, the compounds with weak CDK9 inhibitory activity in the biochemical assay, for example, **1a**, **2d**, and **4d** (IC_50_ of 1.67, 2.03, and 8.5 µM, respectively), also exhibited a weak cytotoxic effect in the MTT assay (average IC_50_ of 437.39, 126.11, and 18.24 µM, respectively). This could indicate that the anticancer mechanism of these compounds is through the inhibition of the CDK9.

The compounds that showed the greatest cytotoxic effect on cancer cell lines were further evaluated for their cytotoxicity on normal non-cancerous cells FHC. The results are shown in Table 3. Compounds **1b**, **3a**, **3c**, and **4a** showed IC_50_ values of 122.51, 195.78, 97.19, and 76.07 µM on normal cells, respectively, and average selectivity over three cancer cells of 43, 41, 15, and 3.5 fold, respectively. This indicates that the most promising compounds displayed selective cytotoxicity effect on cancer cell lines.

#### 2.2.3. Molecular Docking Study into CDK9 Active Site

A docking simulation of **2c**, **3a**, **3c** and **4a** into the ATP binding site of CDK9 was carried out with AutoDock Vina embedded in PyRx 0.8 software using CDK9 co-crystalized with CR8 inhibitor (PDB:3LQ5). The validation of the docking test was performed by re-docking of the co-crystalized ligand (CR8). It revealed a docking score of −8.7 kcal/mol with an RMSD value of 1.7 Å. The key hydrogen bond interactions between the Cys106 and aminopurine ring of CR8 were obtained.

**Figure 5:**
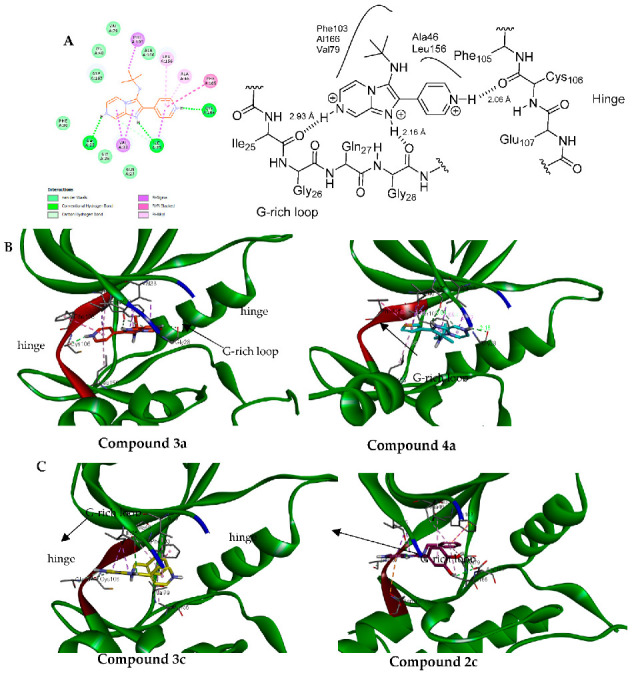
(**A**). Two-dimensional diagram of **3a** showing the key binding residues (left) and schematic representation of **3a** with CDK9 (right). (**B**): proposed binding mode of **3a** (left) and **4a** (right) were the imadazo[1,2-*a*]pyrazine occupies the G-rich loop. (**C**): proposed binding mode of **3c** (left) and **2c** (right) where imadazo[1,2-*a*]pyrazine core interacts with hinge.

Generally, all tested compounds were able to fit into ATP binding pocket and interact well with key amino acid residues in the CDK9 binding site. (Figure 5, Table 4). All docked compounds showed a hydrogen bond interaction between *N*1 of imadazo[1,2-*a*]pyrazine ring and Ile25. Additionally, in all compounds, the alkyl side chain attached to the amino group in position 3 of imadazo[1,2-*a*]pyrazine occupied the hydrophobic pocket in the CDK9 binding site and formed several hydrophobic interactions with Phe103, Val29, Ala166, Ala46, and Leu156.

Compounds **3a** and **4a** showed similar binding pose, were *N*6 of imadazo[1,2-*a*]pyrazine interacted with Gly28 of G-rich loop, and the pyridin-4-yl of **3a** and thiophen-3-yl of **4a** in position 2 of imadazo[1,2-*a*]pyrazine bind to the hinge region by forming a hydrogen bond with Cys106 (Figure 5).

While compounds **2c** and **4c** showed a flipped orientation compared to compounds **3a** and **4a**, a pyrazine ring occupies the hinge region by forming several interactions with Glu107, Asp109, and Cys106, while the dihydroxy phenyl ring of **2c** in position 2 of imadazo[1,2-*a*]pyrazine interacts with G-rich loop and forms two hydrogen bonds with Asp167.

### 2.3. Antiviral Activity

#### 2.3.1. Anti-Coronaviral Inhibitory Activity

The anti-coronaviral activity of the new compounds was assessed using a cytopathic effect (CPE) inhibition assay against human coronavirus 229E. Ribavirin (well-established antiviral agent) was used as standard in this assay.

The results showed that compound **3b** demonstrated good anti-coronaviral activity with an IC_50_ concentration of 56.96 µM; it also exhibited no cytotoxic effect on target cells at concentrations that produced an antiviral effect of 0.1 to 1000 µg/mL, with a 50% cellular cytotoxicity CC_50_ concentration of 406.86 µM and a selectivity index of 7.14. Therefore, **3b** showed better antiviral activity and selectivity than standard drug ribavirin (Table 5).

#### 2.3.2. Anti-Coronavirus Target Identification and Docking Studies

**Figure 6:**
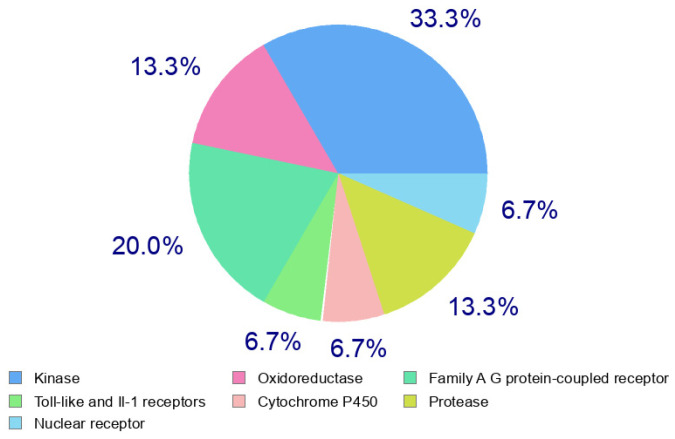
Target predication of compound **3b**.

The predication of target for the viral activity of our compound was carried out using SwissTarget [37]. The data of the target predication of compound **3b** are presented in Figure 6. The result showed that **3b** showed high affinity to protease enzyme as target. Therefore, the potent antiviral activity of **3b** demonstrated in antiviral assay could be due to the inhibition of coronavirus protease.

Human coronavirus (HCoV-229E) shares high homology sequence similarity to coronavirus SARS-CoV-2 but is less pathogenic [38]. Therefore, SARS-CoV-2, which caused acute and lethal respiratory disease in 2019 (COVID-19) was selected for molecular docking studies in this study [39].

Docking studies of **3b** into SARS-CoV-2 main protease enzyme were carried out with AutoDock Vina embedded in PyRx 0.8 software using COVID-19 main protease co-crystalized with X77 inhibitor (PDB:6W63). The validation of the docking test was performed by re-docking the co-crystalized ligand (X77). It revealed a docking score of −9.7 kcal/mol with RMSD value of 1.7 Å. The key hydrogen bond interactions between the Glu166 and Gly143 residues and X77 inhibitor were obtained.

**Figure 7:**
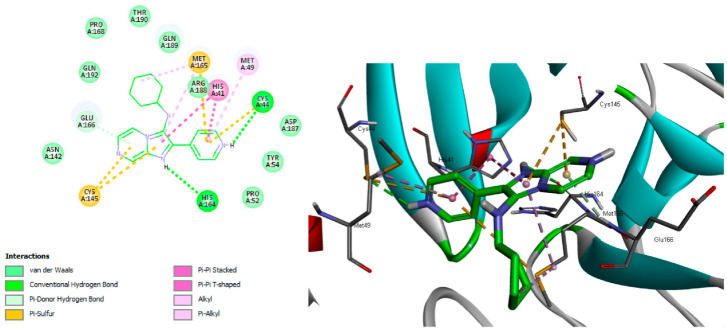
Proposed binding mode of **3b** with COVID-19 main protease.

The results of the docking studies displayed a hydrogen bond interaction between *N*-pyridine of **3b** and Cys44. The imadazo[1,2-*a*]pyrazine of **3b** interacted with Phe140 residue via a hydrogen bond. The pyridyl and cyclohexyl rings of **3b** formed several hydrophobic interactions with Met165, Met49, His41, and Cys44. The imadazo[1,2-*a*]pyrazine also formed hydrophobic interactions with Cy145, Glu166, and His41, as shown in Figure 7 and Table 6.

### 2.4. In Silico Predication of the Compound’s Physiochemical and Pharmacokinetic Properties

#### 2.4.1. Physiochemical and Drug Likeness Properties

Molecular and physiochemical parameters of compounds **1b**, **3c**, and **4a** were calculated using SwissADMET [40] and presented in Table 7. All derivatives exhibited good drug-likeness properties and followed Lipinski role of five with zero violation. Their molecular weight is less than 500; their lipophilicity is within the optimal range (log *P* between 1 and 5); their polarity is within the optimal level (tPSA < 90 Å^2^); and the number of hydrogen acceptors/donor < 5. Therefore, our compounds are expected to be orally bioavailable. Additionally, they demonstrated good aqueous solubility (Log S < −5).

#### 2.4.2. Pharmacokinetic and Toxicity Studies

Pharmacokinetic and toxicity studies of compounds **1b**, **3c**, and **4a** were estimated in silico using the ADMETLab platform [41], and the data of these studies are shown in Table 8. Our compounds demonstrated excellent oral absorption; they showed good caco-2 permeability and low papp efflux (<5.15), none of them are expected to be a substrate or inhibitor of p-glycoprotein, and they all tested positive for human intestine absorption and had a high score of oral bioavailability. They also showed acceptable distribution profiles, with a volume of distribution within the optimal range (0.04–20 L/kg) and reasonable binding to plasma protein within the accepted level (<90%), but all studied derivatives showed high probability to cross the blood–brain barrier. With respect to metabolism, they proved to be inhibitors of CYP3A4, but none of them are expected to be a substrate of the same CYP isoform. They showed excreted T_1/2_ > 1.5 h and clearance lower than 2.5 min/kg/L, which is considered an accepted excretion profile during the drug development process. On the other hand, they showed a suboptimal toxicity profile by demonstrating high probability for the inhibition of hERG, high probability for acute toxicity, and hepatic injury, but they did not cause skin sensitivity at the maximum recommended dose.

## 3. Materials and Methods

### 3.1. Chemistry

Chemicals such as 2-aminopyrazine, aldehydes, and isocyanides were obtained from TCI and Alderich Chemical Companies. The measurement of melting points was conducted using the electrothermal melting point apparatus (UK), and the results were not corrected. NMR spectra data were obtained using Bruker Ascend 700 NMR spectrometer (Fällanden, Switzerland) at 700.17 MHz for proton NMR and 176.08 MHz for carbon NMR, and DMSO-*d*_6_ was used as a solvent in all samples. Thin-layer chromatography (TLC) technique was used as a monitoring technique to check the reaction progression using silica-gel-precoated aluminum sheets (60 F254, Merck) and visualize it under UV at 365 and 254 nm wavelength. The reactions were carried out in a microwave using Biotage, Initiator Sweden AB using vial size 2.0–5.0 mL and normal absorption level.

**General procedure:**

2-Aminopyrazine (0.52 mmol, 1.3 equiv.), required aldehyde (0.4 mmol, 1 equiv.), required isocyanide (0.52 mmol, 1.3 equiv.), and scandium(III)-trifluoromethanesulfonate (0.015 mmol, 10%) were placed in a microwave vial, and then 2 mL of DCM:MeOH (3:1) was added. The reaction mixture then heated in the microwave to 150 °C, low-boiling solvents for 30 min. After cooling to ambient temperature, the reaction solvent was evaporated, and the residue was extracted with DCM (3 × 5 mL). The combined organic layers were dried with MgSO_4_ and then concentrated. The residue was purified by column chromatography using hexane:EtOAc.

*N*-(*Tert*-butyl)-2-phenylimidazo[1,2-*a*]pyrazin-3-amine **(1a)**

Yield: 88.7%; yellow oil. ^1^H NMR (700 MHz, DMSO-*d*_6_) δ ppm 1.01 (s, 9H) 4.83 (s, 1H) 7.35 (t, *J* = 7.42 Hz, 1H) 7.45 (t, *J* = 7.74 Hz, 2H) 7.87 (d, *J* = 4.52 Hz, 1H) 8.21 (d, *J* = 8.17 Hz, 2H) 8.43 (dd, *J* = 4.52, 1.51 Hz, 1H) 8.94 (d, *J* = 1.51 Hz, 1H). ^13^C NMR (176 MHz, DMSO-*d*_6_) δ ppm 30.46, 56.67, 117.68, 126.06, 128.22, 128.33, 128.57, 128.87, 134.93, 135.06, 137.06, 142.99. *m/z* (ESI-MS) [M]^+^ 267.15 (100%), [phenyl]^+^ 78.96 (20%), [M-tert-butyl]^+^ 209.21 (2%)

*N*-Cyclohexyl-2-phenylimidazo[1,2-*a*]pyrazin-3-amine **(1b)**

Yield: 92.3%; white solid. (MP: 145–146 °C). ^1^H NMR (700 MHz, DMSO-*d*_6_) δ ppm 1.05–1.13 (m, 3H) 1.24–1.33 (m, 2H) 1.50 (br. s., 1H) 1.64 (d, *J* = 9.68 Hz, 2H) 1.72 (d, *J* = 12.91 Hz, 2H) 2.83–2.91 (m, 1H) 5.09 (d, *J* = 6.88 Hz, 1H) 7.34–7.37 (m, 1H) 7.48 (t, *J* = 7.74 Hz, 2H) 7.86 (d, *J* = 4.52 Hz, 1H) 8.23 (dd, *J* = 8.28, 1.18 Hz, 2H) 8.38 (dd, *J* = 4.73, 1.51 Hz, 1H) 8.94 (d, *J* = 1.51 Hz, 1H). ^13^C NMR (176 MHz, DMSO-*d*_6_) δ ppm 25.00, 25.78, 34.06, 56.91, 116.85, 127.17, 128.09, 128.17, 128.74, 128.95, 134.27, 136.27, 136.98.142.97. *m/z* (ESI-MS) [M]^+^ 293.22 (100%), [phenyl]^+^ 78.96 (50%), [M-cyclohexyl]^+^ 209.21 (5%).

*N*-Benzyl-2-phenylimidazo[1,2-*a*]pyrazin-3-amine **(1c)**

Yield: 96.1%; yellow oil. ^1^H NMR (700 MHz, DMSO-*d*_6_) δ ppm 4.15 (d, *J* = 6.45 Hz, 2H) 5.73 (t, *J* = 6.35 Hz, 1H) 7.24 (s, 5H) 7.35–7.39 (m, 1H) 7.49 (t, *J* = 7.53 Hz, 2H) 7.76 (d, *J* = 4.52 Hz, 1H) 8.15 (d, *J* = 7.74 Hz, 2H) 8.20 (d, *J* = 4.52 Hz, 1H) 8.89 (s, 1H). ^13^C NMR (176 MHz, DMSO-*d*_6_) δ ppm 51.19, 116.56,127.35, 127.62, 128.19, 12852, 128.73, 129.00, 134.20, 136.17, 136.45, 140.00, 142.33, 142.93. *m/z* (ESI-MS) [M]^+^ 301.13 (100%), [phenyl]^+^ 78.96 (70%), [M-benzyl]^+^ 209.21 (5%).

*N*-(4-Methoxyphenyl)-2-phenylimidazo[1,2-*a*]pyrazin-3-*amine***(1d)**

Yield: 91.0%; yellow oil. ^1^H NMR (700 MHz, DMSO-*d*_6_) δ ppm 3.64 (s, 3H) 6.48 (m, *J* = 9.03 Hz, 2H) 6.77 (m, *J* = 9.03 Hz, 2H) 7.43 (t, *J* = 7.64 Hz, 2H) 7.51 (d, *J* = 3.87 Hz, 1H) 7.86–7.91 (m, 1H) 7.92 (s, 1H) 8.01 (dd, *J* = 4.63, 1.40 Hz, 1H) 8.09 (dd, *J* = 8.39, 1.29 Hz, 2H) 8.12 (s, 1H). ^13^C NMR (176 MHz, DMSO-*d*_6_) δ ppm 55.68, 114.66, 114.88, 115.53, 116.88, 122.88, 127.22, 129.08, 129.63, 133.35, 135.06, 137.51, 138.86, 143.39, 153.10. *m/z* (ESI-MS) [M-Me]^+^ 302.10.

4-(3-(*Tert*-butylamino)imidazo[1,2-*a*]pyrazin-2-yl)benzene-1,2-diol **(2a)**

Yield: 84.1%; white solid. (MP: 270–272 °C). ^1^H NMR (700 MHz, DMSO-*d*_6_) δ ppm 1.03 (s, 9H) 4.65 (s, 1H) 6.77 (d, *J* = 8.17 Hz, 1H) 7.53 (dd, *J* = 8.17, 1.94 Hz, 1H) 7.65 (d, *J* = 2.58 Hz, 1H) 7.82 (d, *J* = 4.52 Hz, 1H) 8.37 (dd, *J* = 4.63, 1.40 Hz, 1H) 8.86 (d, *J* = 1.29 Hz, 1H) 8.96 (br. s., 1H) 9.04 (br. s., 1H). ^13^C NMR (176 MHz, DMSO-*d*_6_) δ ppm 30.54, 56.63, 115.60, 115.97, 117.42, 119.92, 125.03, 126.18, 128.64, 136.79, 141.51, 142.35, 145.29, 145.79. *m/z* (ESI-MS) [M]^+^ 299.10.

4-(3-(Cyclohexylamino)imidazo[1,2-*a*]pyrazin-2-yl)benzene-1,2-diol **(2b)**

Yield: 75.6%; white solid. (MP: 296–298 °C). ^1^H NMR (700 MHz, DMSO-*d*_6_) δ ppm 1.07–1.14 (m, 3H) 1.25–1.32 (m, 2H) 1.51 (br. s., 1H) 1.62–1.67 (m, 2H) 1.71 (d, *J* = 12.26 Hz, 2H) 2.83–2.92 (m, 1H) 4.86 (d, *J* = 6.88 Hz, 1H) 6.80 (d, *J* = 8.17 Hz, 1H) 7.54 (dd, *J* = 8.17, 2.15 Hz, 1H) 7.68 (d, *J* = 2.15 Hz, 1H) 7.81 (d, *J* = 4.73 Hz, 1H) 8.31 (dd, *J* = 4.52, 1.29 Hz, 1H) 8.84 (d, *J* = 1.29 Hz, 1H) 9.00 (s, 1H) 9.07 (s, 1H)^13^C NMR (176 MHz, DMSO-*d*_6_) δ ppm 25.03, 25.84, 34.03, 56.75, 114.92, 115.99, 116.51, 118.78, 125.68, 126.69, 128.76, 136.09, 137.76, 142.26, 145.59, 145.81 *m/z* (ESI-MS) [M]^+^ 325.12 (100%), [cyclohexyl]^+^ 83.10 (10%).

4-(3-(Benzylamino)imidazo[1,2-*a*]pyrazin-2-yl)benzene-1,2-diol **(2c)**

Yield: 84.9%; white solid. (MP: 118–220 °C). ^1^H NMR (700 MHz, DMSO-*d*_6_) δ ppm 4.13 (br. s., 2H) 5.55 (br. s., 1H) 6.84 (br. s., 1H) 7.25 (br. s., 5H) 7.49 (br. s., 1H) 7.64 (br. s., 1H) 7.70 (br. s., 1H) 8.11 (br. s., 1H) 8.81 (br. s., 1H) 9.06 (br. s., 1H) 9.12 (br. s., 1H).^13^C NMR (176 MHz, DMSO-*d*_6_) δ ppm 51.10, 115.08, 116.10, 116.22, 118.93, 125.57, 127.32, 127.58, 128.53, 128.59, 128.71, 135.91, 137.34, 140.19, 142.24, 145.73, 145.92 *m/z* (ESI-MS) 331.06.

4-(3-((4-Methoxyphenyl)amino)imidazo[1,2-*a*]pyrazin-2-yl)benzene-1,2-diol **(2d)**

Yield: 91.4%; white solid. (MP: 150–152 °C). ^1^H NMR (700 MHz, DMSO-*d*_6_) δ ppm 3.64 (s, 3H) 6.44 (d, *J* = 8.82 Hz, 2H) 6.75 (d, *J* = 8.17 Hz, 1H) 6.77 (d, *J* = 8.82 Hz, 2H) 7.39 (dd, *J* = 8.28, 2.04 Hz, 1H) 7.59 (d, *J* = 2.15 Hz, 1H) 7.84 (d, *J* = 4.52 Hz, 1H) 7.92 (dd, *J* = 4.52, 1.51 Hz, 1H) 7.97 (s, 1H) 9.00 (d, *J* = 1.29 Hz, 1H) 7.91 (m, 1H) 7.92 (s, 1H) 8.01 (dd, *J* = 4.63, 1.40 Hz, 1H) 8.09 (dd, *J* = 8.39, 1.29 Hz, 2H) 8.12 (s, 1H). ^13^C NMR (176 MHz, DMSO-*d*_6_) δ ppm 55.69, 114.59, 114.96, 115.50, 116.03, 116.60, 118.91, 120.52, 124.74, 129.40, 137.27, 139.02, 140.69, 142.70, 145.68, 146.37, 152.97 *m/z* (ESI-MS) [M]^+^ 349.23.

*N*-(*Tert*-butyl)-2-(pyridin-4-yl)imidazo[1,2-*a*]pyrazin-3-amine **(3a)**

Yield: 91.3%; dark viscous oil. ^1^H NMR (700 MHz, DMSO-*d*_6_) δ ppm 1.05 (br. s., 9H) 5.00 (br. s., 1H) 7.91 (br. s., 1H) 8.19 (br. s., 2H) 8.47 (br. s., 1H) 8.65 (br. s., 2H) 9.01 (br. s., 1H). ^13^C NMR (176 MHz, DMSO-*d*_6_) δ ppm 30.40, 57.08, 117.94, 122.35, 122.99, 127.76, 129.10, 137.35, 137.81, 143.76, 150.16. *m/z* (ESI-MS) [M]^+^ 268.15*N*-Cyclohexyl-2-(pyridin-4-yl)imidazo[1,2-*a*]pyrazin-3-amine **(3b)**.

Yield: 97.0%; yellow oil.^1^H NMR (700 MHz, DMSO-*d*_6_) δ ppm 1.11 (br. s., 3H) 1.32 (d, *J* = 10.11 Hz, 2H) 1.51 (br. s., 1H) 1.64 (br. s., 2H) 1.75 (d, *J* = 11.83 Hz, 2H) 3.27 (br. s., 1H) 5.27 (br. s., 1H) 7.89 (br. s., 1H) 8.16 (br. s., 2H) 8.41 (br. s., 1H) 8.67 (br. s., 2H) 9.00 (br. s., 1H).^13^C NMR (176 MHz, DMSO-*d*_6_) δ ppm 25.05, 25.72, 34.08, 57.39, 117.09, 121.18, 122.00, 129.14, 130.16, 132.89, 136.63, 143.87, 150.17. *m/z* (ESI-MS) [M]^+^ 294.40 (100%), [M-cyclohexyl]^+^ 211.08 (10%).

*N*-Benzyl-2-(pyridin-4-yl)imidazo[1,2-*a*]pyrazin-3-amine **(3c)**

Yield: 88.6%; yellow oil.^1^H NMR (700 MHz, DMSO-*d*_6_) δ ppm 4.19 (d, *J* = 6.02 Hz, 2H) 5.96 (t, *J* = 6.24 Hz, 1H) 7.23 (s, 5H) 7.80 (d, *J* = 4.09 Hz, 1H) 8.09 (d, *J* = 4.52 Hz, 2H) 8.23 (d, *J* = 4.30 Hz, 1H) 8.67 (d, *J* = 4.30 Hz, 2H) 8.96 (s, 1H).^13^C NMR (176 MHz, DMSO-*d*_6_) δ ppm 51.39, 116.88, 121.43, 127.74, 128.61, 128.78, 130.71, 132.04, 136.49, 139.70, 141.91, 143.84, 149.82, 151.25. *m/z* (ESI-MS) [M]^+^ 302.08 (100%), [M-benzyl]^+^ 211.05 (80%).

*N*-(4-Methoxyphenyl)-2-(pyridin-4-yl)imidazo[1,2-*a*]pyrazin-3-amine **(3d)**

Yield: 95.6%; yellow oil.^1^H NMR (700 MHz, DMSO-*d*_6_) δ ppm 3.66 (br. s., 3H) 6.54 (br. s., 1H) 6.80 (br. s., 1H) 7.89 (br. s., 1H) 7.95 (br. s., 1H) 8.09 (br. s., 1H) 8.37 (br. s., 1H) 8.65 (br. s., 1H) 8.69 (br. s., 2H) 8.83 (br. s., 2H) 9.19 (br. s., 1H). ^13^C NMR (176 MHz, DMSO-*d*_6_) δ ppm 55.71, 115.07, 115.57, 121.58, 123.02, 129.90, 132.03, 132.97, 142.23, 144.36, 149.17, 151.25, 153.42, 156.50. *m/z* (ESI-MS) [M]^+^ 318.04 (100%), [M-Me]+ 302.08 (15%), [M-methoxyphenyl]^+^ 211.05 (10%).

*N*-(*Tert*-butyl)-2-(thiophen-3-yl)imidazo[1,2-*a*]pyrazin-3-amine **(4a)**

Yield: 90.7%; yellow oil.^1^H NMR (700 MHz, DMSO-*d*_6_) δ ppm 1.06 (br. s., 9H) 4.84 (s, 1H) 7.62 (br. s., 1H) 7.85 (br. s., 2H) 8.17 (br. s., 1H) 8.41 (br. s., 1H) 8.91 (s, 1H).^13^C NMR (176 MHz, DMSO-*d*_6_) δ ppm 30.49, 56.69, 117.70, 124.03, 125.54, 126.18, 128.02, 128.87, 135.91, 137.02, 138.13, 142.73. *m/z* (ESI-MS) [M]^+^ 273.10.

*N*-Cyclohexyl-2-(thiophen-3-yl)imidazo[1,2-*a*]pyrazin-3-amine **(4b)**

Yield: 85.6%; white solid. (MP: 170–172 °C). ^1^H NMR (700 MHz, DMSO-*d*_6_) δ ppm 1.09–1.15 (m, 3H) 1.29–1.33 (m, 2H) 1.51–1.54 (m, 1H) 1.64–1.68 (m, 2H) 1.75 (d, *J* = 12.48 Hz, 2H) 2.85–2.95 (m, 1H) 5.04 (d, *J* = 6.67 Hz, 1H) 7.66 (dd, *J* = 4.95, 2.80 Hz, 1H) 7.82–7.84 (m, 1H) 7.86 (d, *J* = 4.52 Hz, 1H) 8.10 (d, *J* = 2.80 Hz, 1H) 8.34 (dd, *J* = 4.52, 1.29 Hz, 1H) 8.89–8.94 (m, 1H). ^13^C NMR (176 MHz, DMSO-*d*_6_) δ ppm 25.07, 25.80, 34.14, 56.98, 116.90, 123.08, 126.76, 127.19, 127.35, 128.76, 135.03, 135.47, 136.27, 142.47. *m/z* (ESI-MS) [M]^+^ 299.12.

*N*-Benzyl-2-(thiophen-3-yl)imidazo[1,2-*a*]pyrazin-3-amine **(4c)**

Yield: 89.3%; yellow oil.^1^H NMR (700 MHz, DMSO-*d*_6_) δ ppm 4.17 (d, *J* = 6.02 Hz, 2H) 5.65 (t, *J* = 6.13 Hz, 1H) 7.20–7.29 (m, 5H) 7.66 (br. s., 1H) 7.75 (d, *J* = 3.87 Hz, 1H) 7.79 (d, *J* = 4.73 Hz, 1H) 8.06 (br. s., 1H) 8.11–8.15 (m, 1H) 8.87 (s, 1H). ^13^C NMR (176 MHz, DMSO-*d*_6_) δ ppm 51.30, 116.59, 123.06, 126.81, 127.23, 127.64, 127.82, 128.63, 128.76, 134.42, 135.38, 136.17, 140.08, 142.67. *m/z* (ESI-MS) [M]^+^ 307.04 (100%), [M-benzyl]^+^ 216.02 (30%).

*N*-(4-Methoxyphenyl)-2-(thiophen-3-yl)imidazo[1,2-*a*]pyrazin-3-amine **(4d)**

Yield: 88.6%; yellow oil.^1^H NMR (700 MHz, DMSO-*d*_6_) δ ppm 3.64 (br. s., 3H) 6.48 (d, *J* = 7.10 Hz, 2H) 6.77 (d, *J* = 7.31 Hz, 2H) 7.63 (d, *J* = 2.58 Hz, 1H) 7.66–7.69 (m, 1H) 7.89 (br. s., 1H) 7.95 (br. s., 1H) 8.03 (br. s., 1H) 8.06 (br. s., 1H) 9.06 (br. s., 1H). ^13^C NMR (176 MHz, DMSO-*d*_6_) δ ppm 55.69, 114.69, 115.52, 116.85, 121.26, 123.77, 126.93, 127.14, 129.64, 134.73, 137.33, 137.47, 138.95, 143.10, 153.12. *m/z* (ESI-MS) [M-Me]^+^ 307.07 (100%), [M-methoxyphenyl]^+^ 2016.02 (20%).

### 3.2. In Vitro CDK9 Assay

The CDK9 assay kit was purchased from BPS Biosciences (San Diego, CA, USA) Catalogue# 79628, and the anti-CDK effect was measured following the manufacturer’s instructions using kinase-GLO^®^ MAX as a detection component. The luminescence was measured using a microplate reader. The results were analyzed using GraphPad Prism 5.0, and the kinase inhibitory activity was expressed as IC_50_ for three individual experiments. In the assay, dinaciclib was used as a positive control, and DMSO was used as negative control.

### 3.3. MTT Cytotoxicity Assay

The in vitro cytotoxicity of our compounds was determined using MTT assay. Cancer cells were exposed to different concentrations of the compounds, ranging from 0.1 to 10 µM for 48 h. The treated cells were then incubated with 10% *v*/*v* reconstituted MTT for 3 h before reading the plates on Wallac Victor2 1420 multilabel counter in fluorescence mode at a wavelength of 460/590 nm (ex/em). The results were analyzed using GraphPad Prism 5.0, and cytotoxicity was expressed as IC_50_ for three individual experiments. In the assay, staurosporine was used as a positive control, and DMSO was used as a negative control.

### 3.4. Antiviral Assay

The crystal violet method was used to evaluate antiviral activity and cytotoxicity assays using cytopathic (CPE) inhibition effect as described by Choi et al., 2009 [42]. In this assay, the low pathogenic coronavirus (229E) was used. The percentage of antiviral activities of the test’s compounds were calculated according to Pauwels et al. (1988) [43] using the following equation: antiviral activity = [(mean optical density of cell controls − mean optical density of virus controls)/(optical density of test − mean optical density of virus controls)] × 100%. Based on these results, the 50% CPE inhibitory dose (IC50) was calculated. The results were analyzed using GraphPad Prism 5.0, and cytotoxicity was expressed as IC_50_ for three individual experiments. In the assay, ribavirin was used as a positive control, and DMSO was used as a negative control.

### 3.5. Docking Studies

The ligands used in these studies (compounds **2c**, **3a**, **3b**, **3c**, and **4a**) were prepared using ChemBoiDraw Ultra 14.0, and each ligand file was saved as PDB format. The crystal structure of the proteins was obtained from Protein Data Bank (CDK9 co-crystalized with CR8 inhibitor; PDB:3LQ5 and COVID-19 co-crystalized with X77 inhibitor; PDB:6W63), and each protein file was downloaded as a PDB file. Then, the preparation of the protein was performed using Discovery studio by selecting one subunit of the protein and inverted the selection and remove other molecules. The clean protein was then saved as a PDB file. The co-crystalized ligand was also saved as a PDB file and used in the docking studies as a comparative reference in determining the binding pocket. Then, the AutoDock software was used to introduce polar hydrogens to the protein, and it was saved as PDBQT file. The docking studies was conducted using AutoDock vina and grid box embedded in PyRx software. The docking results were analyzed using Discovery Studio by the visualization of the ligand–receptor interactions.

## 4. Conclusions

In summary, novel imadazo[1,2-*a*]pyrazines were synthesized and evaluated for their anticancer and antiviral activities. All the newly synthesized compounds were evaluated in a CDK9 inhibitory assay as the molecular target for their anticancer activity. The data of CDK9 biochemical assay showed that the derivatives with pyridin-4-yl in position 2 of imadazo[1,2-*a*]pyrazine **3(a**–**d)** displayed the most potent CDK9 inhibitory activity with IC_50_ range 0.16–0.8 µM. The cytotoxicity effect of all synthesized compounds was evaluated in an MTT assay against breast cancer (MCF7), colorectal cancer (HCT116), and chronic myelogenous leukaemia (K652) cell lines. The results of this assay showed that the cytotoxic effect of the inhibitors is correlated to their inhibitory activity against CDK9. Compounds **3c**, **3a**, and **1b** showed the most potent cytotoxicity effect, with average IC_50_s of three cell lines of 0.16, 0.26, and 0.25 µM, respectively. The new compounds demonstrated reasonable physiochemical and predicated pharmacokinetic properties including lipophilicity, aqueous solubility, absorption, distribution, and elimination. The most promising agents were evaluated in an antiviral assay against human coronavirus (HCoV-229E). In this assay, **3b** displayed the most potent antiviral activity, with an IC_50_ of 56.96 µM and selectivity index of 7.14, better than the standard drug ribavirin. The target predication result revealed that **3b** showed high affinity to protease enzyme. Therefore, the potent anti-coronaviral in in vitro assay could be contributed to the inhibition of protease enzyme. Docking studies of **3b** with COVID-19 main protease were conducted and showed good binding affinity, which supports the in vitro results.

## Figures and Tables

**Table 1:** CDK9 inhibitory activity in biochemical assay of compound **1a**–**4d**. Data are presented as mean ± standard deviation of three individual experiments.

Compound	CDK9 Inhibitory Activity IC_50_ (µM)
**1a**	1.67 ± 0.025
**1b**	0.25 ± 0.004
**1c**	1.01 ± 0.017
**1d**	1.04 ± 0.019
**2a**	1.14 ± 0.019
**2b**	0.89 ± 0.016
**2c**	0.31 ± 0.006
**2d**	2.03 ± 0.04
**3a**	0.26 ± 0.004
**3b**	0.41 ± 0.007
**3c**	0.16 ± 0.003
**3d**	0.8 ± 0.014
**4a**	0.73 ± 0.011
**4b**	1.32 ± 0.006
**4c**	0.71 ± 0.012
**4d**	3.16 ± 0.058
**Dinaciclib**	0.08 ± 0.002

**Table 2:** Cytotoxicity effect of compound **1**–**4** in MTT assay. Data are presented as means ± standard deviation of three individual experiments.

Compound	IC_50_ (µM)		
	MCF7	HCT116	K652
**1a**	405.48 ± 5.05	596.96 ± 8.25	309.74 ± 4.49
**1b**	3.01 ± 0.04	1.43 ± 0.02	50.27 ± 0.8
**1c**	98.95 ± 1.39	59.3 ± 5.05	88.62 ± 8.25
**1d**	243.71 ± 3.62	75.23 ± 1.24	77.44 ± 1.34
**2a**	76.75 ± 1.08	35.86 ± 0.56	22.79 ± 0.37
**2b**	119.3 ± 1.82	105.43 ± 1.78	54.25 ± 0.96
**2c**	39.71 ± 0.62	59.87 ± 1.04	88.62 ± 1.09
**2d**	80.37 ± 1.31	161.61 ± 2.41	136.35 ± 2.59
**3a**	4.3 ± 0.5	2.69 ± 0.04	46 ± 0.67
**3b**	31.7 ± 0.44	8.96 ± 0.14	67.83 ± 1.08
**3c**	8.59 ± 0.12	7.96 ± 0.12	4.45 ± 0.06
**3d**	117.96 ± 1.17	27.91 ± 1.88	46.56 ± 0.48
**4a**	25.37 ± 0.32	61.31 ± 0.12	12.77 ± 0.06
**4b**	180.96 ± 2.63	27.91 ± 0.43	46.58 ± 0.76
**4c**	55.81 ± 0.8	80.61 ± 1.26	69.19 ± 3.89
**4d**	141.13 ± 2.13	115.39 ± 1.93	87.16 ± 1.53
**Staurosporine**	18.41 ± 0.4	10.86 ± 0.26	22.08 ± 0.56

**Table 3:** Cytotoxicity effect of compound **1b**, **3a**, **3c**, and **4a** against normal cells FHC. Data are presented as mean ± standard deviation of three individual experiments.

		SI		
Compound	FHC IC_50_ (µM)	MCF7	HCT116	K652
**1b**	122.51 ± 19.1	40.70	85.67	2.40
**3a**	195.78 ± 2.79	45.53	72.78	4.25
**3c**	97.19 ± 1.58	11.31	12.21	21.84
**4a**	76.07 ± 1.11	3.04	1.2	6.3
**Staurosporine**	38.19 ± 0.97	2.07	3.5	1.7

SI = Selectivity index (IC_50_ in normal cells FHC/IC_50_ in cancer cells.

**Table 4:** Docking results of **3a**, **4a**, **3c**, and **2c** into the ATP binding site of CDK9.

Compound	Docking Score (Kcal/mol)	Interaction Residue (Type of Interaction)	Bond Length (Å)
**3a**	−8.6	Cys106 (HB)	2.06
		Ile25 (HB)	2.93
		Gly28 (HB)	2.16
		Phe105 (Pi-Pi stacked)	5.29
		Phe103 (Pi-sigma)	3.82
		Val33 (Pi-sigma)	3.54 and 3.71
		Leu156 (Pi-alkyl)	4.60
		Ala46 (Pi-alkyl)	4.51
**4a**	−8.7	Ile25 (HB)	3.01
		Gly28 (HB)	2.16
		Phe103 (Pi-Pi stacked)	3.69
		Phe105 (Pi-Pi stacked)	4.16
		Ala46 (pi-alkyl)	3.51
		Ala166 (pi-alkyl)	5.43
		Leu156 (pi-alkyl)	3.69
**3c**	−8.3	Ile25 (HB)	3.09
		Phe103 (PI-Pi stacked)	4.22
		Ala46 (pi-alkyl)	4.52
		Ala166 (pi-alkyl)	4.81
		Val79 (pi-alkyl)	5.31
		Val33 (pi-sigma)	4.93
**2c**	−8.5	Asp167 (HB)	2.62 and 2.47
		Ile25 (HB)	2.59
		Asp109 (Pi-anion)	4.98
		Val33 (pi-alkyl)	4.66
		Phe103 (Pi-pi stacked)	4.41
		Ala166 (pi-alkyl)	4.75
		Val79 (pi-alkyl)	5.46
		Ala56 (pi-alkyl)	4.71

**Table 5:** 229E inhibitory activity in biochemical assay of compound **1d**, **3a**, **3b**, and **3c**. Data are mean ± standard deviation of three individual experiments.

Compound	CC50 (µM)	IC50 (µM)	SI
**1b**	299.32	145.92	2.051
**3a**	940.19	393.66	2.39
**3b**	406.86	56.96	7.14
**3c**	471.51	379.45	1.24
**Ribavirin**	160.47	113.81	1.4

SI = selectivity index (CC_50_/IC_50_).

**Table 6:** Docking study results of **3b** with COVID-19 main protease.

Docking Score (Kcal/mol)	Interaction Residue (Type of Interaction)	Bond Length (Å)
**−7.6**	His164 (HB)	2.96
	Cys44 (HB)	2.91
	Cys145 (pi-sulfur)	5.11, 5.13
	Met165 (pi-sulfur)	2.91
	Met49 (Pi-alkyl)	5.16
	His41 (Pi-Pi stacked)	4.38, 5.45

**Table 7:** Calculated molecular and physiochemical parameters of compound **1b**, **3c**, and **4a**.

Compound	M. W	Clog *P*	tPSA (Å^2^)	Solubility Log *S*	HBA	HBD	Lipinski
**1b**	292.38	3.24	42.22	−4.78	2	1	Yes, 0 violation
**3c**	301.35	2.68	55.11	−4.07	3	1	Yes, 0 violation
**4a**	272.37	2.82	70.46	−3.98	2	1	Yes, 0 violation

**Table 8:** Estimated ADMET properties of compounds **1b**, **3c**, and **4a**. Red color means unfavorable property.

Compound			1b	3c	4a
Property	Test	Recommended			
**Absorption**	Papp (Caco-2 permeability)	>−5.15	−4.595	−4.86	−4.472
	Pgp-inhibitor		No	No	No
	Pgp-substrate		No	No	No
	HIA (human intestinal absorption)		+	+	+
	Bioavailability score	0.55	0.55	0.55	0.55
**Distribution**	PPB (Plasma protein binding)	<90%	82.523	87.219	72.632
	BBB (Blood–brain barrier)		+	+	+
	VD (volume of distribution)	0.04–20 L/kg	0.698	0.542	0.29
**Metabolism**	CYP3A4 inhibitor		Yes	Yes	Yes
	CYP3A4-substrate		No	No	No
**Excretion**	T_1/2_ (Half live)	>0.5 h	2.014	1.774	1.918
	Clearance	<15 mL/min/kg	2.063	1.952	1.489
**Toxicity**	hERG blocker		Yes	Yes	No
	Ames mutagenicity		+	+	+
	Skin sensitization		No	No	No
	LD_50_ of acute toxicity	>500 mg/kg	2.59	2.596	2.59
	DILI (drug induce liver injury)		+	+	+
	FDAMDD (maximum recommended daily dose)		+	+	+

## Data Availability

Data is contained within the article.
